# PP2A Phosphatase as a Regulator of ROS Signaling in Plants

**DOI:** 10.3390/antiox5010008

**Published:** 2016-03-02

**Authors:** Moona Rahikainen, Jesús Pascual, Sara Alegre, Guido Durian, Saijaliisa Kangasjärvi

**Affiliations:** 1 Department of Biochemistry, Molecular Plant Biology, University of Turku, FI-20014 Turku, Finland; moona.rahikainen@utu.fi (M.R.); sara.alegregarcia@utu.fi (S.A.); guido.durian@utu.fi (G.D.); 2 Plant Physiology Lab, Organisms and Systems Biology, Faculty of Biology, University of Oviedo, 33006 Oviedo, Spain; pascualjesus@uniovi.es

**Keywords:** plant, reactive oxygen species (ROS), signaling, protein phosphorylation, Protein Phosphatase 2A (PP2A)

## Abstract

Reactive oxygen species (ROS) carry out vital functions in determining appropriate stress reactions in plants, but the molecular mechanisms underlying the sensing, signaling and response to ROS as signaling molecules are not yet fully understood. Recent studies have underscored the role of Protein Phosphatase 2A (PP2A) in ROS-dependent responses involved in light acclimation and pathogenesis responses in *Arabidopsis thaliana*. Genetic, proteomic and metabolomic studies have demonstrated that trimeric PP2A phosphatases control metabolic changes and cell death elicited by intracellular and extracellular ROS signals. Associated with this, PP2A subunits contribute to transcriptional and post-translational regulation of pro-oxidant and antioxidant enzymes. This review highlights the emerging role of PP2A phosphatases in the regulatory ROS signaling networks in plants.

## 1. Introduction

Plants are frequently challenged by changing weather conditions and the presence of detrimental biotic stress factors, including microbial pathogens and insect herbivores. To cope with the ever-changing natural circumstances, plants have evolved mechanisms to sense and respond to the external factors to optimize their fitness. To achieve this, stress-exposed plant tissues must decide whether it is advisable to sacrifice the tissue through a tightly regulated process of programmed cell death, or whether it is more beneficial to invest in energy-consuming acclimation processes in order to ensure seed production. The “choice” is the outcome of signal integration from multiple sources, and deploys signaling pathways elicited by reactive oxygen species (ROS) in different cellular compartments [1,2]. Analysis of cross-communication among different pathways is a modern trend in plant biology [3]. This review focuses on the emerging role of PP2A phosphatases as a regulator of antioxidant activities and ROS signaling in plants.

During the past decade, intensive research has demonstrated that ROS signaling is a common factor in biotic and abiotic stress responses. In biotic interactions, recognition of external factors by plasma membrane receptor kinases leads to activation of plasma membrane NADPH oxidases causing an ROS burst in the apoplast, with a consequent activation of phosphorylation-relay cascades that trigger the first line of defense gene expression in the nucleus [4,5,6]. Abiotic stresses in turn are generally considered to involve alterations in organellar redox biology and ROS signaling, albeit the exact mechanisms remain unsolved [2]. Various combinations of light stress, heat and drought are, however, well known to promote increased formation of ROS, and the photosynthetic machinery is generally considered the major site of this ROS production [7,8,9,10]. While harsh abiotic stress conditions may cause oxidative damage to the photosystems [11,12], studies have demonstrated that ROS-induced signals also essentially regulate the expression of nuclear-encoded genes responsible for defensive measures and antioxidant functions, thereby triggering acquired stress resistance in aboveground organs [10,13,14,15,16].

**Figure 1:**
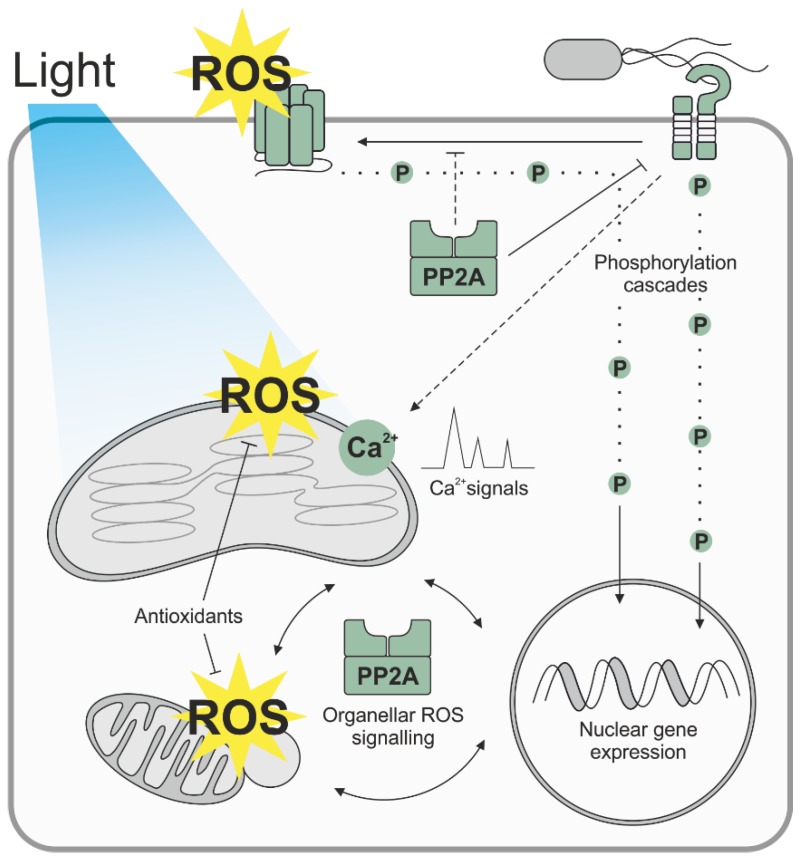
Protein Phosphatase 2A as a regulator of ROS signaling in plant leaves. In plant biotic interactions, recognition of conserved microbial structures, such as bacterial flagellin, by plasma membrane receptor kinases leads to NADPH oxidase-driven ROS burst in the apoplast and activation of phosphorylation-relay cascades that trigger defence gene expression in the nucleus. Abiotic stresses, such as light stress, in turn promote alterations in organellar redox biology and ROS signaling, which also modulate nuclear gene expression and stress resistance in green plant tissues. Recognition of attempted infection is also rapidly relayed into the chloroplasts, where Ca^2+^-dependent signaling interactions are needed to trigger additional signaling events, which further modulate immunity-related gene expression in the nucleus. PP2A protein phosphatase limits the activity of the plasma membrane receptor kinase BAK1, and, consequently, the flagellin-induced ROS burst into the apoplast. Additionally, PP2A modulates photo-oxidative stress responses by controlling organellar ROS signaling and the abundance of alternative oxidases in leaf mitochondria.

More recently, the role of organellar ROS signaling in plant biotic interactions has also been recognized [17,18]. Recognition of a presence of a pathogen in the apoplast is rapidly relayed into the chloroplasts, where Ca^2+^-dependent signaling interactions are needed to trigger additional signaling events, which further modulate immunity-related gene expression in the nucleus ([19,20], Figure 1). The importance of chloroplast ROS burst in plant immunity is also reflected by a recent report by Zabala *et al.* [21], who showed that the bacterial pathogen *Pseudomonas syringae* secretes pathogen effectors that are targeted to chloroplasts, where they prevent the organellar ROS burst by modulating the function of the photosynthetic electron transfer chain.

Even though it is evident that plants can sense the chemical nature (superoxide, hydrogen peroxide, singlet oxygen and hydroxyl radical) and sub-cellular localization of ROS, and trigger defense reactions accordingly, the signaling networks determining the acclimation response remain poorly understood [22]. Even so, it is clear that evolution of mechanisms to sensitively respond to ROS as signaling molecules has provided significant competitiveness to plants in different ecological niches.

Since redox regulation is a key component in plant acclimation to varying light conditions, particularly in high light stress, light-dependent changes in gene expression may partially overlap with those induced by pathogens [2]. Besides light intensity, the duration of the photoperiod has proved to be a key component that modulates both the availability of ROS [23] as well as plant responses to elevated ROS levels [24,25,26]. Yet another layer of cross-communication is commonly observed as acquired cross-tolerance. Plants pre-exposed to high levels of light, for example, display increased resistance to virulent strains of the bacterial hemibiotrophic pathogen *Pseudomonas syringae* or green peach aphid (*Myzus persicae*) [27,28]. Such cross-tolerance commonly associates with enhanced production of ROS and consequent oxidative signaling [29]. To keep the oxidative signals in balance, plants deploy a multi-layered antioxidant network, including enzymes such as catalases (CAT), superoxide dismutases (SOD), ascorbate peroxidases (APX), peroxiredoxins (PRX) and thioredoxins (TRX) as well as the low-molecular-weight antioxidants ascorbate and glutathione, which maintain ROS balance and metabolic homeostasis in different cellular compartments [30].

Beside antioxidant activities, post-translational regulation that modulates the activation state of signaling proteins is an important level of regulation in ROS signaling. Reversible phosphorylation of signaling proteins is an evolutionarily conserved mechanism that drives phosphorylation-relay cascades and their signaling interactions in animals and plants. Controlled activities among mitogen-activated protein kinases (MPKs) and Ca^2+^-dependent protein kinases (CDPKs, or CPKs in *Arabidopsis thaliana*) are well known for their importance in cellular stress signaling [31,32]. Here, we focus on the emerging role of type 2A protein phosphatases in these interactions.

## 2. PP2A Phosphatase as a Regulatory Enzyme in Plant Stress

Trimeric type 2A protein phosphatases, composed of a catalytic subunit C, scaffold subunit A and regulatory subunit B, are evolutionarily conserved signaling components that regulate stress signaling in both animals and plants [33]. The regulatory B subunit is commonly referred to as the “specificity unit” that determines the target specificity of the trimeric PP2A holoenzyme [33,34]. The *Arabidopsis thaliana* genome contains five different genes encoding C subunits, three genes for the A subunits and 17 genes encoding the variable regulatory B subunits, which are further divided into B, B′ and B’’ families [35]. The trimeric holoenzyme compositions therefore provide extensive variability and versatility for PP2A in regulatory networks. Besides the regulatory B subunit, PP2A function is further regulated by reversible methylation of a conserved C-terminal leucine residue [36]. Moreover, both animals and plants possess regulatory components that further modulate the phosphatase activity as has been shown e.g., for the regulatory TAP46 protein in *Arabidopsis* [37].

Recent studies have assigned functions for PP2A in plant stress signaling in several different model crop species. The potato PP2A catalytic subunit StPP2Ac2 and the tomato subunits LePP2Ac1 and LePP2Ac2 mediate jasmonic acid signaling after wounding [38], while the tomato subunits LePP2Ac1, LePP2Ac2 and LePP2Ac3 operate in cold stress [38]. Studies have also shown that the rice PP2A catalytic subunit OsPP2A-1-5 as well as the potato subunits StPP2Ac1, StPP2Ac2a, StPP2Ac2b and StPP2Ac3 become transcriptionally upregulated under salinity stress [38,39,40]. Moreover, in wheat, TaPP2Ac-1 acts as a positive regulator of salt stress responses [41]. Most of the research efforts have focused on unraveling the roles of the catalytic C-subunits, whereas little is known about the regulatory B-subunits. In wheat, B′′α subunit has been shown to positively regulate lateral root formation under osmotic stress conditions [42]. Identification and characterization of other PP2A subunits in crop species will help in interpreting how PP2A regulates abiotic stress responses in useful plants.

In the model plant *Arabidopsis thaliana*, a recessive mutation of the catalytic subunit PP2A-C2 leads to enhanced sensitivity to abscisic acid (ABA), suggesting that this subunit is a negative regulator of ABA-mediated responses [43]. The scaffold subunit PP2A-A1, or ROOTS CURL IN NAPHTHYLPHTHALAMIC ACID1 (RCN1), in turn acts as a positive regulator in ABA-related pathways, and *rcn1* mutants additionally display pleiotropic phenotypes in other phytohormone signaling pathways as well [44,45]. PP2A-A3 has been identified as an important element in the low-temperature signaling pathway in *Arabidopsis*, inhibiting the cold adaptative response [46]. Regarding the regulatory B-subunits, B′′α and B′′β have been shown to modulate plant isoprenoid biosynthesis pathway, B′′α playing a distinct role in plants under salt stress [47]. The regulatory subunit PP2A-B′γ, in turn, seems to be present in heterotrimers involved in heat stress response [48]. A *pp2a-b′γζ* double mutant, deficient in two highly similar subunits B′γ and B′ζ showed increased resistance towards photo-oxidative stress under a stress-combination of high light, elevated temperature and limited water availability [49]. Hence, different PP2A subunits, presumably operating in different trimeric PP2A holoenzymes, mediate both positive and negative regulation of abiotic stress responses, allowing delicate fine-tuning of responses under a range of environmental challenges.

PP2A has also been shown to function in plant-biotic interactions. Catalytic PP2A subunits of tobacco have been related to effector-triggered immunity (ETI) [50], a defense response which commonly includes a hypersensitive response, a particular type of cell death in plants. In *Arabidopsis*, the subunit B′γ negatively regulates defense against green peach aphids and is required for transcriptional and post-translational control of immune processes [28,51]. Moreover, mutants deficient in B′γ and B′θ were shown to display lowered susceptibility towards the bacterial pathogen *Pseudomonas syringae pv. tomato* [52]. Mechanistic insights into PP2A-dependent regulation were recently provided by Segonzac *et al.* [53], who showed that a trimeric PP2A protein phosphatase composed of the catalytic subunit C4, the scaffold subunit A1 and regulatory B subunits B′η or B′ζ binds and limits the autophosphorylation, and hence activity, of the plasma membrane receptor kinase BAK1. These protein interactions also negatively regulate the flagellin-induced ROS burst into the apoplast [53]. Consequently, *pp2a-c4* and *pp2a-a1* knock-out lines are more resistant to the virulent bacterial pathogen *Pseudomonas syringae pv. tomato* [53]. Taken together, PP2A subunits respond to and mediate various stress signaling effects in different plant species, but the molecular mechanisms are only starting to emerge.

PP2A mutant lines with deficiencies in the regulation of stress responses may show simultaneous changes both in hormone and ROS signaling. This stems from the fact that ROS signaling and hormonal signaling pathways are tightly connected, not only in eliciting stress resistance but also in determining developmental processes [54]. ROS act as secondary messengers in many hormone-signaling pathways, but, in the case of stress signaling, ROS work also upstream by eliciting hormonal signals. In *Arabidopsis*, PP2A-B′γ has been shown to be a negative regulator of organellar ROS accumulation and the consequent salicylic acid (SA) signaling elicited by these ROS [55]. In ABA signaling, PP2A-B′α, B′β and B′δ are direct interactors of the protein kinase OPEN STOMATA 1 (OST1), which is a major component of ABA signaling in guard cells and has been shown to regulate apoplastic ROS production through phosphorylation of the NADPH oxidase RbohF [56,57]. However, the physiological role of PP2A in these interactions remains unclear. Altogether, further studies are needed to pinpoint the specific regulatory nodes in the stress signaling networks governed by PP2A.

## 3. PP2A-B′γ as a Regulator of ROS Signaling and Cell Death

Attempts to understand the role of PP2A in stress resistance led to the identification of PP2AB′γ as a putative component in light acclimation and defense signaling in *Arabidopsis* [51]. When grown under 50% humidity and moderate light intensity, *pp2a-b′γ* mutant displayed premature yellowing and constitutive activation of salicylic acid-(SA-) and jasmonic acid-(JA-) dependent pathogenesis responses, whereas growth under high levels of light abolished the yellowing phenotype and also the immune reactions become alleviated. Notably, even slight variations in the growth conditions, such as growth under 65% humidity at 200 µmol·m^−2^·s^−1^ [50] was sufficient to rescue the yellowing *pp2a-b′γ* mutant phenotype, which makes it a tricky tool for analysis of PP2A mediated phenotypes by mutant approaches. The yellowing *pp2a-b′γ* mutant phenotype was accompanied by elevated foliar ROS levels and eventually cell death under moderate light intensities, but significant alterations in the redox status of the main antioxidants ascorbate and glutathione were not observed [51,55,58].

Recently, by taking advantage of selective reaction monitoring (SRM) mass spectrometry, Konert *et al.* [58] showed that *pp2a-b′γ* may partially circumvent the constitutive ROS accumulation through a feedback loop where ROS induce up-regulation of the mitochondrial bypass pathways employing alternative oxidases AOX1A and AOX1D. AOX1A has been extensively studied and shown to operate in diverting excess reducing equivalents and hence in minimizing the formation of ROS within the photosynthetic and mitochondrial electron transfer chains [59,60]. The other AOX isoforms have remained less well characterized and their importance in stress responses is not well understood. Shuttling of redox-active intermediates between the organelles and the cytoplasm has a very likely impact on the formation of ROS in different cellular compartments and hence modulate redox signaling effects within the entire cell [59,61].

To promote organellar ROS signaling effects and to stabilize the *pp2a-b′γ* mutant phenotype, Li *et al.* [55] took advantage of the *catalase 2* (*cat2*) mutant as a tool to trigger oxidative signaling in the *pp2a-b′γ* mutant background. CATALASE 2 (CAT2) is the major antioxidant enzyme that quenches photorespiratory H_2_O_2_ in peroxisomes, and hence provides a highly informative system to study factors involved in intracellular ROS signaling [62]. Intriguingly, however, SA-induced cell death of *cat2* is conditioned by day length and becomes observable only under long-day conditions, even though oxidative stress in the *cat2* mutant prevails also under short photoperiods [24,25,63]. Recently, Li *et al.* [55] showed that, under short photoperiods, the ROS-induced cell death becomes suppressed through pathways that require the activity of PP2AB′γ. This conclusion was supported by proteomic analysis, which showed that PP2A-B′γ is required to control ROS-induced changes in the abundance and phosphorylation of the key pathogenesis-associated marker proteins PATHPGENESIS-RELATED PROTEIN 2 (PR2) and PATHOGENESIS-RELATED PROTEIN 5 (PR5) in short-day conditions [55].

Even though PP2A-B′γ is required to control intracellular oxidative stress responses and associated cell death in *Arabidopsis* [55], analysis of PP2A-B′γ and the highly similar PP2A-B′ζ in stress responses revealed that deficiencies in these PP2A subunits lead to alleviation of biotic stress responses and induction of photoprotective mechanisms and enhanced tolerance against abiotic stress [28,49]. Hence, PP2A-B′γ and PP2A-B′ζ appear to mediate opposing effects on cell death regulation and impact the decision between ROS-induced cell death and acclimation [49]. However, the underlying mechanisms remain to be established, and hence what determines the threshold is not well understood. It has been suggested that the ultimate outcome of ROS-induced responses, which may lead to activation of a strictly controlled cascade of programmed cell death or elicit protective mechanisms and acclimation, becomes determined by opposing actions of pro-cell death and anti-cell death signaling mechanisms [22]. Intriguingly, the outcomes of the genetic interactions in the *cat2 pp2a-b′γ* double mutant and in the *pp2a-b′γζ* are very different. While salicylic acid signaling and cell death are promoted in the *cat2 pp2a-b′γ* double mutant that experiences oxidative stress [55], in the double mutant combination *pp2a-b′γζ*, protective photo-oxidative stress responses are activated at an increased level, resulting in an enhanced activation of light acclimation [49]. Signaling in plant cells is characterized by interplay between different signal transduction pathways, often also such diverse ones as biotic and abiotic stress response pathways. PP2A-B′γ and PP2A-B′ζ control intracellular ROS homeostasis and signaling and thereby influence the plants different physiological outcomes, comprising cell death, growth and acclimation upon environmental perturbations.

## 4. PP2A as a Regulator of Antioxidant Activities

Proteomic studies suggested that PP2A-B′γ is required to control the abundance of antioxidant enzymes, such as the chloroplastic copper/zinc SOD 2 (CSD2) and monodehydroascorbate reductase 2 (MDAR2), as well as the mitochondrial AOX1A and 1D, all of which have well-known roles in the maintenance of cellular ROS homeostasis [51,55,58]. The AOX1A gene is a target for ANAC013-mediated transcriptional activation in response to mitochondrial stress signals [64]. ANAC013 is an important regulator of mitochondrial retrograde regulation/signaling, since it controls a set of genes that are common and robust candidates for mitochondrial retrograde regulation under different types of stresses (denoted Mitochondrion Dysfunction Stimulon Genes) [64]. Indeed, Giraud *et al.* [65] showed that AOX1A is required for full tolerance against combined effects of light stress and drought.

While the *pp2a-b′γ* mutant may partially circumvent ROS accumulation through enhanced quenching of redox equivalents by mitochondrial AOX activity [58], transcript profiling of high light acclimated *pp2a-b′γζ* double mutants did not show up-regulation of AOX1A gene expression [49]. Instead, *pp2a-b′γζ* double mutants displayed increased transcript abundance for the H_2_O_2_ scavenging enzyme ascorbate peroxidase 2 (APX2), heat shock transcription factor A3 (HSFA3) and the heat shock proteins HSP18.2, HSP21 and HSP22, which are transcriptionally co-regulated targets of HSFs [49]. The APX2 gene is transcriptionally highly responsive to a number of factors, including reduction state of the photosynthetic electron transfer chain, ABA signaling, chloroplastic and apoplastic ROS, and metabolic signals exemplified by accumulation of phosphoadenosine 5′-phosphate (PAP) in stress-exposed leaves [13,66,67,68]. Jung *et al.* [69] suggested, that HSFA3 would modulate APX2 expression under high levels of light and high temperature but would not mediate signals arising from reduction of the plastoquinone pool in the photosynthetic thylakoid membranes. A good candidate for mediating the HSFA3-dependent activation of APX2 is H_2_O_2_, which is generated in both heat- and light-induced stresses [70]. Through a yet unidentified mechanism, PP2A-B′γ is required to control the extent of photo-oxidative stress responses, exemplified here by enhanced expression of APX2 in *Arabidopsis* leaves.

## 5. Conclusions

PP2A phosphatases appear to control multiple targets in the stress signaling networks in plants. Such modulation of different signaling nodes is likely to involve PP2A phosphatases with different heterotrimeric compositions. Besides resolving the identity of the regulatory PP2A holoenzymes and the functional redundancy among individual PP2A subunits, the key research questions still to be resolved include elucidation of the PP2A target phosphoproteins and their functional significance and co-operation in the stress resistance in plants.

## Abbreviations

The following abbreviations are used in this manuscript:

ABA	abscisic acid
AOX	alternative oxidase
APX	ascorbate peroxidases
CAT	catalase
CDPK, CPK	calcium-dependent protein kinase
CSD	copper/zinc superoxide dismutase
ETI	effector-triggered immunity
H_2_O_2_	hydrogen peroxide
HSF	heat shock factor
HSP	heat shock protein
JA	jasmonic acid
MDAR	monodehydroascorbate reductase
MPK	mitogen activated protein kinase
PAP	phosphoadenosine 5′-phosphate
PP2A	Protein Phosphatase 2A
PRX	peroxiredoxin
ROS	Reactive oxygen species
SA	salicylic acid
SOD	superoxide dismutase
TRX	thioredoxin
